# Preprocedural fasting for contrast-enhanced CT: when experience meets evidence

**DOI:** 10.1186/s13244-021-01131-1

**Published:** 2021-12-04

**Authors:** Heng Liu, Yu Liu, Li Zhao, Xue Li, Weiguo Zhang

**Affiliations:** 1 Department of Radiology, PLA Rocket Force Characteristic Medical Center, No. 16 Xinjiekou Outer Street, Beijing, 100088 China; 2 Department of Radiology, Daping Hospital, Army Medical University, No. 10 Changjiang Road, Yuzhong District, Chongqing, 400042 China; 3 Beijing Advanced Innovation Center for Big Data-Based Precision Medicine, School of Biological Science and Medical Engineering, Beihang University, Beijing, 100191 China; 4 Chongqing Clinical Research Center for Imaging and Nuclear Medicine, Chongqing, 400042 China

**Keywords:** Preparatory fasting, Contrast-enhanced CT, Nausea, Vomiting, Aspiration pneumonia

## Abstract

Traditional preparatory fasting policy prior to iodinated contrast media (ICM) assisted contrast-enhanced CT (CECT) examinations lacks methodologically acceptable evidence. Considering the possible negative effects of preprocedural fasting, the latest European Society of Urogenital Radiology guidelines V10.0 and American Committee of Radiology 2021 guidelines clearly state that preprocedural fasting is not recommended prior to routine intravenous ICM administration. This comprehensive and detailed Review presents the current global dietary preparation policies, potential harm of excessive fasting, and a systematical and well-bedded description of practice advancements of dietary preparation. The evidences revealed that there has been no single instance of vomiting-associated aspiration pneumonia due to the undemanding implementation of preparatory fasting prior to CECT yet. Non-fasting would not increase the incidence of emetic symptoms and the risk of aspiration pneumonia. Not every patient should undergo all CECT examinations without preparatory fasting. There is still much more refinement to be done on the preparatory fasting policy. Changes in traditional preparatory fasting policy will make positive and significant implications on clinical practice. This Review aims to provide operational guidance and suggestions for practitioners and policymakers, motivate efficient, reasonable, safe and normative ICM usage, and achieve optimal patient clinical benefits and high-quality radiological care practices.

## Key points

- Traditional preparatory fasting policy prior to CECT lacks methodologically acceptable evidence.
- Excessive fasting may bring a series of negative effects on patients.
- Non-fasting will not increase the risk of aspiration pneumonia and the incidence of emetic complications.
- Not every patient should undergo all CECT examinations without preparatory fasting.
- Changes in current preparatory fasting policy will make significant implications on clinical practice.

## Introduction

With the increased availability of CT imaging equipment and rapid advancements of contrast enhancement technology, iodinated contrast media (ICM) assisted contrast-enhanced CT (CECT) examination has become one of the most dominating diagnostic procedures in radiology departments worldwide. CECT increases the image contrast and diagnostic information, and improves the detection rate of lesions, especially tiny or small lesions. According to the different enhancement patterns and degrees of different lesions, CECT is helpful to the diagnosis and differential diagnosis, and shows great clinical application value [1]. As a time-honored tradition, patients are typically instructed to fast for 4–6 h prior to CECT in the majority of medical institutions throughout the world [2]. Preoperative fasting is defined as a prescribed period of time before a procedure when patients are allowed eating and drinking nothing by mouth [3]. The original intention was to prevent emetic complications (e.g., nausea and vomiting) and possible aspiration after ICM administration. Historically, Mendelson reported preoperative fasting in obstetric patients receiving general anesthesia in 1946 [4], and first introduced the concept of pulmonary acid aspiration syndrome, which indicated inhalation of gastric fluid contents induced lung damage. Preoperative fasting was thought to reduce the risk and severity of regurgitation of gastric contents and pulmonary acid aspiration syndrome during anesthesia, although the latter was extremely rare and often non-fatal [5]. For the next 70 years, this preparatory fasting regimen has been gradually promoted as a routine preparation protocol, which is regularly applied in various diagnostic and therapeutic procedures that require general anesthesia, local anesthesia, procedural sedation and analgesia, and even prior to CECT which require intravenous injection of ICM. According to the updated American Society of Anesthesiologists (ASA) 2017 guidelines, patients should fast for 2 h, 6 h, and 8 h from clear fluids, light meal and high-fat solids prior to general anesthesia to prevent possible aspiration pneumonia [6]. Very recently, using evidence-based factors and a consensus-derived algorithm, graded fasting precautions for liquids and solids are recommended for elective procedural sedation by International Committee for the Advancement of Procedural Sedation [7].

The initial driving factor of preparatory fasting orders prior to CECT was prompted on a logical assumption that practitioners and policymakers believed that vomiting is more likely to occur when the stomach cavity is full [8]. Fasting is to accelerate gastric emptying as much as possible, and to prevent aspiration of gastric contents and subsequent aspiration pneumonia when patients receive high-osmolality contrast media (HOCM) in a supine position. In the era of first-generation ionic HOCM, the frequency of nausea and vomiting was up to 4.58% and 1.84%, respectively [9]. On the other hand, once severe life-threatening events occur, empty stomach and unobstructed airway can contribute to the convenient implementation of emergency tracheal intubation and airway manipulation. Given this, it seemed necessary, reasonable, and meaningful to conduct preparatory fasting in the past. Despite the fact that there is substantially minimal conclusive and direct scientific evidence to support this practice, and current preparatory fasting policies regarding content and duration prior to CECT lack standardization and are highly heterogeneous across medical institutions and countries (ranging from no fasting to overnight fasting), preparatory fasting remains a universal and common request worldwide [2, 10].

With the introduction and extensive applications of nonionic ICM, ionic HOCM has been gradually phased out from the market. Nowadays, almost all industrialized countries only use nonionic low-osmolality contrast media (LOCM) and iso-osmolality contrast media (IOCM). The classification and physicochemical properties of ICM are shown in Table 1. The manufacturers of nonionic ICM declare that, except for adequate hydration, no other special preparation procedures are required prior to CECT. Nonionic ICM has been shown to reduce the frequency of nausea and vomiting to 0.013% and 0.059% respectively [11]. The literature estimates of the incidence of emetic symptoms are shown in Table 2. Up to now, no case of aspiration pneumonia has been identified due to the undemanding compliance with preparatory fasting prior to CECT yet. Therefore, current overfocus on preparatory fasting may be probably misguided, which may only give practitioners and policymakers false sense of security when holding fasting orders. Its necessity and rationality are increasingly concerned and questioned by clinical departments and radiology departments. It would not seem prudent to fast without distinction for every patient without evidence-based considerations. It is important to note that preparatory fasting has known negative effects on patients, including, but are not limited to, general discomfort (e.g., irritability, dehydration), and hypoglycemic risk in diabetic patients. The latest European Society of Urogenital Radiology (ESUR) guidelines and American Committee of Radiology (ACR) guidelines clearly state that fasting is not recommended prior to routine intravenous ICM administration [33, 34]. In other words, the most frequent dietary protocols, sometime considered a "mandatory preparation regimen", are arbitrary and unjustified, and the existing fasting strategies can reasonably be less restrictive. Nevertheless, as currently practiced, some institutions appear to be reluctant to "deviate from the fasting dogma", attributed to their reliance on deep-rooted clinical experience patterns and lack of evidence-based knowledge.

**Table 1:** Classification and physicochemical characteristics of ICM

Classification	Structure	Generic name	Brand name	Molecular weight	^a^Iodine content	^b^Osmotic pressure	^c^Viscosity
First generation (HOCM)	Ionic monomer	Ditriazoate	Meglumine diatrizoate	809	306	1530	5.0
Second generation (LOCM)	Nonionic monomer	Iohexol	Ominpaque	821	300, 350	680, 830	6.3, 10.4
		Iopamidol	Iopamiro	777	300, 370	616, 796	4.7, 9.4
		Iopromide	Ultravist	791	300, 370	590, 770	4.7, 10.0
		Ioversol	Optiray	807	320, 350	710, 790	5.8, 9.0
		Iobitridol	Xenetix	835	300, 350	695, 915	6.0, 10.0
		Iomeprol	Iomeron	777	300, 400	521, 726	
	Ionic dimer	Ioxaglate	Hexabrix	1270	320	600	7.5
Third generation (IOCM)	Nonionic dimer	Iodixanol	Visipaque	1550	270, 320	290, 290	5.8, 11.8
		Iotrolan	Isovist	1626	300	290	

HOCM, high-osmolality contrast media; LOCM, low-osmolality contrast media; IOCM, iso-osmolality contrast media

^a^mg I/mL

^b^mOsm/kg H_2_O

^c^mPa.s/37℃

**Table 2:** Literature estimates of the incidence of nausea and vomiting symptoms in CECT examinations

Author (year) (ref)	Country	Total subjects	Data collection	Iodinated contrast medium used	Incidence (%)	
					Nausea	Vomiting
Katayama et al. (1990) [9]	Japan	337,647	Prospective	Ionic iodinated contrast medium	4.58%	1.84%
				Non-ionic iodinated contrast medium	1.04%	0.36%
Oowaki et al. (1994) [12]	Japan	2414	Prospective	Amidotrizoic acid	6.7%	
				Iopamidol, Iohexol	1.4%	
Geeter et al. (1994) [13]	Germany	198	Randomized phase III clinical trial	Iomeprol	2%	3%
				Iopromide	3%	2%
Federle et al. (1998) [14]	USA	452	Prospective	Ioversol	1.99%	/
Morteléet et al. (2005) [15]	USA	29,508	Prospective	Iopromide	0.034%	
Nagamoto et al. (2006) [16]	Japan	945	Prospective	Iopamidol	0.85%	/
Wendt-Nordahl et al. (2006) [17]	Germany	49,975	Prospective (PMS)	Iobitridol	0.3%	< 0.1%
Vogl et al. (2006) [18]	Germany	52,057	Prospective (PMS)	Iobitridol	0.24%	0.086%
Vijayalakshmi et al. (2007) [19]	United Kingdom	1985	Prospective	Iopamidol	0.39%	0.29%
				Iomeprol	0.73%	0.52%
Kopp et al. (2008) [20]	Multinational	74,717	Prospective (PMS)	Iopromide	0.54%	
Gomi et al. (2010) [21]	Japan	8931	Prospective	Iopromide, Iomeprol, Iopamidol, Iohexol, Ioversol	0.3%	
Häussler et al. (2010) [22]	Germany	9515	Prospective (PMS)	Iodixanol	0.12%	0.08%
Maurer et al. (2011) [23]	Germany	160,639	Prospective (PMS)	Iobitridol	0.20%	0.06%
García et al. (2013) [24]	Spain	110,041	Retrospective	Iopromide, Iomeprol	0.047%	
Pradubpongsa et al. (2013) [25]	Thailand	55,286	Retrospective	/	0.166%	
Zhang et al. (2013) [26]	China	20,185	Prospective (PMS)	Iodixanol	0.154%	0.089%
Müller (2014) [27]	Germany	10,354	Prospective (PMS)	Iodixanol	0.18%	0.05%
Palkowitsch et al. (2014) [28]	Multinational	132,012	Prospective (including PMS)	Iopromide	0.52%	
Li et al. (2015) [29]	China	109,255	Retrospective	Iopromide, Iodixanol, Iopamidol, Iohexol, Ioversol	0.053%	
Zhang et al. (2016)[30]	China	137,473	Retrospective	Iopromide, Iopamidol	0.023%	0.041%
Li et al. (2018) [11]	China	110,836	Prospective	Iopromide, Iodixanol, Iopamidol, Ioversol, Iobitridol, Iohexol	0.013%	0.059%
Kim et al. (2018) [31]	Korea	1175	Prospective	Iobitridol, Iohexol, Iomeprol, Iversol, Iopamidol	2.9%	0%
Ha et al. (2020) [32]	Korea	864	Retrospective	Iobitridol, Iohexol, Iomeprol, Ioversol, Iopamidol	0.69%	1.39%

None of these studies reported a single instance of vomiting-associated aspiration pneumonia

PMS, post-marketing surveillance

**Fig. 1:**
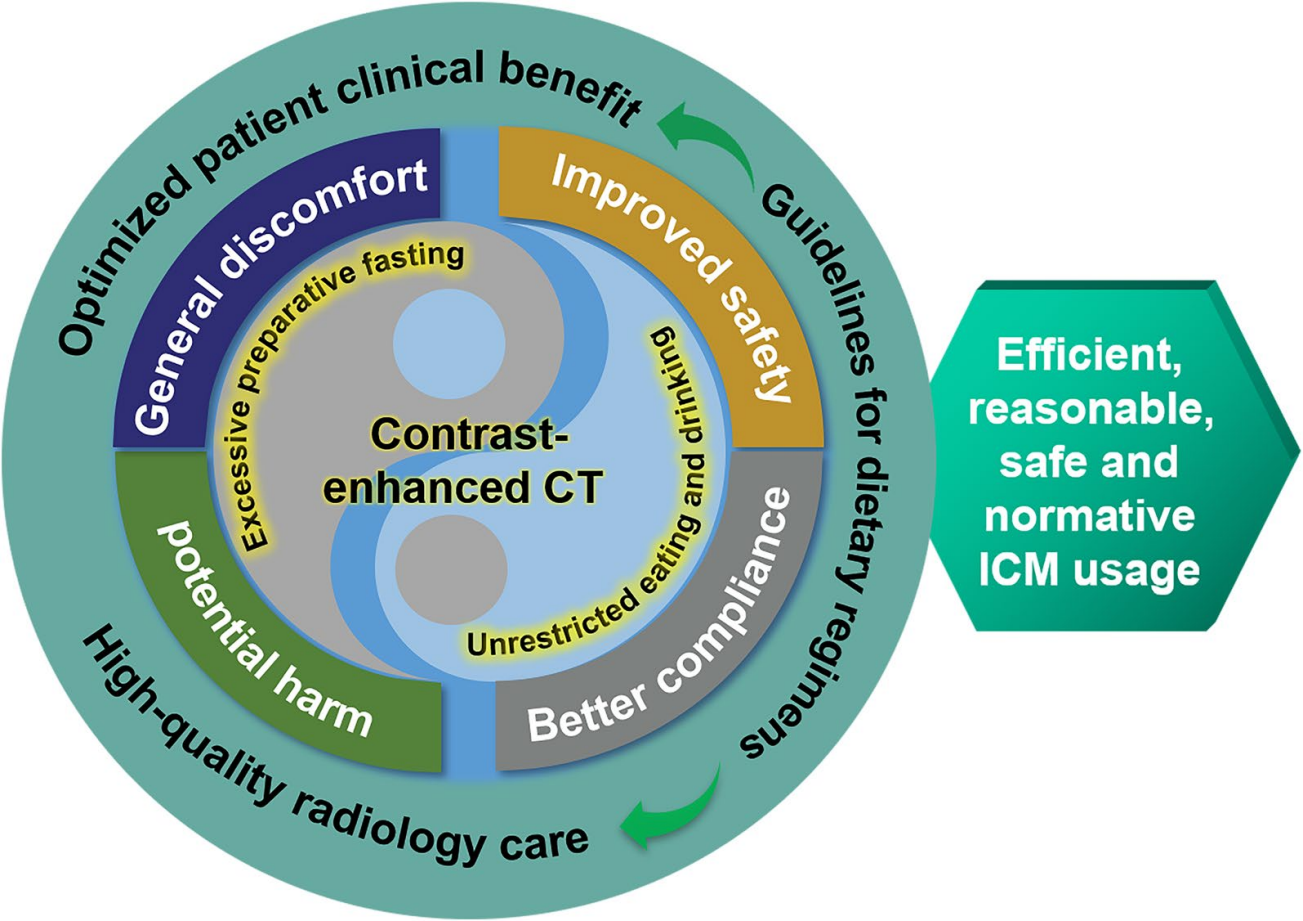
The negative effects of excessive preprocedural fasting and the benefits of non-fasting prior to CECT

This panoramic Review starts with an overview of emetic and aspiration symptoms. It then presents the current status of global dietary preparation policies prior to CECT and potential harm of excessive fasting, with a comprehensive and detailed description of the state-of-the-art clinical practice advancements of dietary preparedness. Finally, it discusses the existing issues and prospects possible future striving directions. This review aims to deepen the understanding of the dietary preparedness prior to CECT, provide operational guidance and suggestions for practitioners and policymakers, motivate efficient, reasonable, safe and normative ICM usage, and achieve optimal patient clinical benefits and high-quality radiological care practices (Fig. 1).

## Possible predisposing factors for emetic symptoms

Nausea and vomiting are collectively known as emetic complications. There are a variety of possible predisposing factors for nausea and vomiting occurrence [35], include, but are not limited to: (i) Mental factors: Some patients are extremely anxious, nervous, and even scared about their own conditions. In this scenario, vomiting is easy to occur prior to CECT. (ii) Pharmacological effects: Patients receiving rapid intravenous ICM administration may experience transient oral metal odor, which usually lasts for 10–15 s and then disappears naturally. Patients who are sensitive to peculiar smells may develop transient nausea and vomiting. This is more frequent in HOCM, and most symptoms undergo spontaneous remission without treatment. (iii) Primary diseases: for patients suffering from digestive system infectious diseases, visceral pain diseases, central nervous system diseases, neurogenic vomiting, hypoglycemia, and patients who are under chemotherapy [36, 37], vomiting symptoms may appear or intensify after ICM administration. (iv) Other factors: The causal factors and severity of vomiting is related to overeating or drinking liquids (e.g., sour milk and soda beverages) that produce acid and gas.

It is worth noting that emetic symptoms are often infrequent, transient and self-limited, and vomiting does not necessarily mean pulmonary aspiration, which is defined as inhalation of oropharyngeal or gastric contents into the larynx and lower respiratory tract [38]. Whether in theory or practice, aspiration is more likely to appear in patients with dangerous status such as impaired consciousness, increased intracranial pressure, posterior cranial nerve injury, weakened swallowing reflex, and tracheal intubation. For conscious and cooperative patients, aspiration can be readily avoided simple motions such as turning the head, vomiting, and swallowing. Undoubtedly, the outcome and related risks of aspiration in fully conscious patients during CECT are almost certainly low than those under general anesthesia. It is generally accepted that at least 25 mL (pH = 2.5, > 0.4 mL kg^−1^) of liquid aspiration are required to induce clinically significant lung damage [39]. The volume of gastric contents and the pH of gastric cavity are key factors that affect the consequences of aspiration [40]. Studies showed that prolonging the fasting duration prior to CECT could increase the incidence and severity of emetic complications [12]. Note that fasting does not guarantee an empty stomach. Fasting for more than 3 h not only could not further reduce the volume of stomach contents, but also could lower the pH of gastric cavity [41]. This would increase the risk of aspiration pneumonia. For patients who developed aspiration, the consequences might be more severe.

## Current status of global dietary preparation policies prior to CECT

Lee et al. conducted a multinational survey on the time interval of preparative fasting protocols prior to elective non-gastrointestinal CECT, obtaining responses from 69 institutions in 6 countries [2]. The results showed that the fasting policies highly heterogeneous among different countries, and even among different institutions in any same country, ranging from no fasting to overnight fasting. The fasting duration for solids in the United States, Korea, and Egypt were 0–6 h, 0–8 h, and 0 h-overnight, respectively, and the fasting duration for liquids were 0–4 h, 0–8 h, and 0–6 h in these three countries, respectively [2]. Of the 49 hospitals that implemented solids fasting, the fasting duration for solids were longer than liquids in 20 hospitals [2]. In another multinational survey investigation by Han et al. [10], among 58 Korean hospitals surveyed, 57 instructed patients to fast, of which 33 instructed fasting from both solids and water, with a median fasting time of 6 h. Among 15 Korean hospitals surveyed, 12 instructed patients to fast, of which 10 instructed fasting from only solids, with a median fasting time of 4 h [10].

ESUR Guidelines V10.0 in 2018 stated that fasting was not recommended prior to injection of nonionic LOCM or IOCM [33]. Very recently, ACR 2021 guidelines deliberately added a new chapter for the fasting policy prior to intravenous ICM injection [34]. It recommended that fasting was not required prior to routine intravascular injection of ICM. However, for patients who receiving sedation and anesthesia, the American Society of Anesthesiology guidelines should be consulted [42]. In China, according to the Practice of Medical Imaging Technology Guidelines, the fasting duration for patients receiving non-gastrointestinal examinations and gastrointestinal examinations were 4 h and 4–8 h, respectively [43]. As currently practiced, water can serve as a negative oral contrast agent for gastrointestinal examinations [44, 45]. CT examination technology expert consensus recommended that fasting for at least 4 h was required for gastric enhancement scan [1], and about 800–1000 mL water intake was encouraged prior to gastric and small intestinal examinations. The purpose was to ensure good filling of gastrointestinal tract, to provide universally recognized quality images, and to avoid misdiagnosis and missed diagnosis [46]. Furthermore, patients are strongly encouraged to ingest fluid intravenously or orally (called hydration) hour after hour from 6 to 12 h prior to CECT and within 24 h after examinations [47], to prevent possible contrast-induced nephropathy in high-risk patients [48]. However, for outpatients and emergency patients, intravenous hydration is impractical and oral hydration is more easily available, making the preparatory fasting policy contradictory.

## The potential harm of excessive fasting

The types and contents of solids and liquids consumed must be taken into consideration when determining an appropriate fasting duration. Evidences on gastric emptying investigations showed that clear liquids containing particles smaller than 2 mm were emptied in an exponential manner, with 10–20 min for 50% emptying, 1 h for 90% emptying, and 2 h for almost complete emptying, respectively [40]. The overall gastric emptying rate of milk in infants and children under 5 years of age is ≥ 80% within 3 h [49]. The emptying rate of solids varies greatly, with 5 h for 50% emptying and about 12 h for complete emptying, respectively. Consuming fatty or fried foods or meat may lead to prolonged time for the stomach to empty. Patients with weakened bowel movement or damaged gastrointestinal function require longer empty time [3].

Although we have obtained the approximate stomach emptying time for different foods, and have recognized the ineffectiveness of traditional fasting regimens [50, 51], some difficulties remain in implementing the new dietary preparation instructions in clinical practice, such as unnecessary fasting or excessive fasting. The reality is that practitioners and policymakers are reluctant to "break the fasting dogma", attributed to their reliance on deep-rooted clinical experience patterns. Some of them even mistakenly believe that the longer the fasting period, the safer the patient. If they deviate from existing guidelines, they are likely to be challenged and face with criticism. In many medical institutions, to avoid risks and responsibilities, many inpatients are near-universally asked to fast substantially exceeding the recommended fasting thresholds by guidelines [52], because they are usually scheduled for multiple radiology and laboratory examinations on the same day. If a patient is scheduled for CECT in the next morning, it has become time-honored tradition to restrict the consumption of liquids and solids from after dinner the day before. This extreme practice may seem to increase the scheduling flexibility of the radiology department on the next day. However, for individual patients, the experience and comfort are greatly reduced, and a series of additional harm are likely to occur.

Excessive fasting can increase the patients’ stress responses into a catabolic state, disrupt the body's internal environment and metabolic balance, and increase the risk of ADR after ICM injection [53–55]. Patients are more susceptible to irritability and anxiety during the waiting or examination process, presented as poor compliance and noncooperation. For elderly patients with malnutrition and weak constitution, they are particularly vulnerable to physical discomfort (e.g., palpitation, thirst, hunger, exhaustion), muscle weakness, hypoglycemia, hypotension, and dehydration symptoms [51, 56–58]. In addition, patients usually choose to stop routine medications during fasting period, which may aggrandize the health risks for patients with diabetes, hypertension, and those who need continuous medication. For patients with special needs, such as diabetic patients, strict fasting from liquids and solids is difficult [51]. For infants and children, excessive fasting can adversely affect metabolism and behaviors [40, 59, 60], leading to irritability and noncooperation [61], particularly susceptible to dehydration and hypoglycemia [62]. This would increase the risk of emetic symptoms [63]. Therefore, these patients will not benefit from any period of fasting. Withholding unreasonable fasting orders and improving patients’ cognitive ability of dietary preparation may increase patient benefits, manage patient turnaround time in the radiology department more effectively. This is significant to save medical resource costs and improve the quality of the radiological care practice.

## Practice progresses of dietary preparation prior to CECT

Given doubts about the necessity and rationality on preparatory fasting, some preliminary attempts and explorations have been made. Very early, Wagner et al. first reported no statistical difference in the frequency of emetic complications between patients who did not fast and those who fasted for at least 4 h [64]. Similarly, Park et al. observed 122 adult patients undergoing abdominal CT examinations due to acute abdominal pain, and found no significant difference in the frequency of emetic complications between patients who did not fast and those who fasted for more than 6 h [65]. In another study by Oowaki et al. [12], preparatory fasting could increase the incidence of emetic complications for HOCM, while not for LOCM. Emetic complications were more frequent in patients who fasted for a prolonged time. In 2012, Lee et al. conducted a rigorous systematic review of 13 studies through Medline [2]. No aspiration was identified in any of 2001 patients who ingested clear inert liquids within 1 h prior to CECT, indicating that fluids ingestion should not be restricted.

In our own practice, since June 2015, we have abolished traditional fasting instructions prior to routine CECT examinations in our institution. Unless compulsory for clinical treatment needs and gastrointestinal examinations, liquids and solids are not restricted in other routine examinations. For urgent or emergency CECT examinations, no delay is based on fasting time. During the next 7 years, more than 450,000 cases of CECT examinations have been performed. Neither increased incidence of emetic complications no case of aspiration pneumonia was observed [11, 29, 66]. These interesting findings suggest that withholding fasting orders does not offer any additional harm for patients, which should be verified in longer observation period and more other institutions. Very recently, a variety of clinical investigations such as prospective clinical observations, retrospective analysis, historical controlled trials, and prospective randomized controlled trials, have been reported, which provided valuable clinical references for reevaluating current preparatory fasting policy (Table 3). These studies attempt to answer five specific questions that need to be addressed: (i) Must all patients fast? (ii) Which population need to fast? (iii) What is the most appropriate fasting duration? (iv) What is the specific fasting content? (v) What harm may occur after fasting?

**Table 3:** Examples of preparative fasting policies prior to CECT in literature

Author (year)(ref)	Country	Data collection	Total subjects	Patients age	Iodinated contrast medium used	Food Preparation Policy	
						Fasting group	Non-fasting group
Wagner et al. (1997) [64]	France	Prospective	1000	59 ± 15	Iopromide	Fast from liquids and solids for at least 4 h	Unrestricted consumption of liquids and solids
Park et al. (2008) [65]	Korea	Clinical observation	122	> 15	–	Fast for < 6 h	Fast for ≥ 6 h
Li et al. (2018) [11]	China	Prospective	110,836	0–104	Iopromide, Iodixanol, Iopamidol, Ioversol, Iobitridol, Iohexol	Fast from solids for 4 h	Unrestricted consumption of liquids and solids
Kim et al. (2018) [31]	Korea	Prospective	1175	20–91	Iobitridol, Iohexol, Iomeprol, Ioversol, Iopamidol	Fast from solids for 6 h	
Barbosa et al. (2018) [67]	Brazil	Randomized controlled study	3206	18–97	Ioversol	Fast for at least 4 h	Receive a light meal (juice, jelly, biscuits, and small sandwiches)
Ha et al. (2020) [32]	Korea	Retrospective	864	0–20	Iobitridol, Iohexol, Iomeprol, Ioversol, Iopamidol	For patients who required oral sedation or who did not require sedation, solid food and non-clear liquids were restricted: (i) < 12 months: fast from for 2 h;1 to 4 years: fast for 3 h; ≥ 5 years: fast for 4 h For patients who require intravenous sedation: all ages: fast from clear liquids, breast milk, and solid food for 2 h, 4 h, and 6 h, respectively	
Tsushima et al. (2020) [68]	Japan	Historical control trial	57,973	0–99	Iopromide, Iomeprol, Iopamidol, Iohexol, Ioversol	Fast one meal (restrict solid intake)	Unrestricted consumption of liquids and solids
Neeman et al. (2021) [69]	Israel	Randomized controlled study	2091	18–98	Iopromide	Fast for at least 4 h	Unrestricted consumption of liquids and solids

### Prospective clinical observations

Li et al. first conducted a prospective observation in a large-scale population who underwent routine CECT [11]. Among them, 51,807 cases fasted from solids for 4 h, and 59,029 cases were not restricted from solids, and free liquids consumption was allowed for all patients. The results showed an extremely low total incidence of emetic complications (0.071%), and no case of aspiration was identified. Such a low incidence was attributed to the standardized pre-inspection screening and evaluation process implemented by the author's institution. There was no statistical difference in the frequency of emetic complications between the two groups of patients with different basic data. The patients with an ICM-ADR history (1.04%) had a higher incidence of emetic complications than those with other ADR histories (0.12%) and those without ADR history (0.06%). They concluded that there was no association between the solid preparedness and the occurrence of emetic complications. Patients with an ICM-ADR history should be given particular attention to reduce possible adverse events.

In another study, Kim et al. evaluated the effect of fasting duration on the occurrence of emetic complications in 1175 adult patients [31]. The patients received instructions for preparatory fasting for 6 h from solids. As a result, 34 cases (2.9%) developed mild nausea after receiving LOCM and no case of vomiting was identified. The actual median fasting duration for solids and liquids were 14 h and 11 h, respectively. The length of fasting time from solids and liquids were not related to the nausea severity, indicating that a considerable proportion of patients experienced unnecessary excessive fasting longer than the instructed interval. Furthermore, they identified that drug allergy history (excluding ICM hypersensitivity) and iobitridol usage were independent risk and protective factors for nausea respectively, with an odds ratio of 4.33 and 0.32, respectively.

### Retrospective analysis on pediatric patients

Infants and children have high nutritional requirements, strong metabolism, and low glycogen storage, representing a special patient population that requires special attention. Excessive fasting can detrimentally affect their metabolism and behaviors [40, 59, 60, 62], leading to irritability, noncooperation, and especially prone to dehydration and hypoglycemia. Currently, the vast majority of studies involving preprocedural fasting in children focus on the fields of surgery and anesthesia, and preparatory fasting prior to radiological procedures mostly follows the fasting schemes adopted in these fields [70]. Sedation is often required for some children to control their behaviors and movement, to ensure the high quality of the scanned images. According to the latest anesthesia guidelines, for infants and children who require sedation, clear fluids, breast milk, and solids should be restricted for 2 h, 4 h, and 6 h, respectively (named 6–4–2 fasting regimen) [60, 63, 70]. There are no standard preparatory fasting recommendations for children who do not require sedation yet.

Ha et al. evaluated the incidence of emetic complications and their risk factors after intravenous LOCM administration in pediatric patients [32]. For patients who required oral sedation or who did not require sedation, the fasting duration from solids and non-clear liquids depended on their ages. The fasting duration in infants under 12 months, children that were 1–4 years old, and children that were 5–20 years old were 2 h, 3 h, and 4 h, respectively, without restriction on clear liquids ingestion (e.g., water and tea without carbohydrates). For patients who require intravenous sedation, the aforementioned 6–4–2 fasting regimen was implemented regardless of their ages. The results showed that of the evaluated 864 patients, 18 cases (2.1%) developed emetic complications, with a low incidence of severe vomiting (0.22%), and no case of aspiration pneumonia was identified. 57.2% of patients experienced unnecessary excessive fasting. The average fasting duration in patients with and without emetic complications were 7.9 h and 8.7 h, respectively, indicating no correlation between fasting time interval and the occurrence of emetic complications. Further analysis showed that ongoing chemotherapy, iomeprol, and iohexol usage were independent risk factors, with odds ratios of 4.323, 7.219, and 5.241, respectively.

### Historical control trials

The ESUR guideline version 10.0 released in 2018 clearly states that preparatory fasting is not recommended prior to nonionic LOCM or IOCM administration [33]. To follow this new statement, Tsushima et al. abolished the traditional one-meal fasting policy, announcing in-house that solids were no longer forbidden prior to CECT [68]. The effect of policy changes on the incidence of acute ADR was analyzed. The results showed no significant difference in the overall frequency of acute ADR (1.6% vs 1.4%) and severe allergy-like reactions (0.06% vs 0.05%) before and after the policy change (1.4%). After abolishing the fasting instructions, the incidence of nausea (0.18% vs 0.31%) and vomiting (0.16% vs 0.12%) decreased significantly and remained unchanged respectively, and no case of aspiration pneumonia was identified. Considering that the decreased frequency of nausea may decline patient discomfort, the historical control results suggested that there was no need for preparatory fasting prior to CECT.

### Prospective randomized controlled trials

CECT is indispensable significance for rational tumor theranostics, including preoperative diagnosis and staging, formulation of treatment plans, evaluation of treatment responses, and follow-up review. Tumor patients are often accompanied with other major chronic diseases due to their poor physical conditions. The long-term radiotherapy and the radiation superposition effect of multiple CECT scans contributed to increased ADR risk following ICM administration. In addition, some anti-tumor drugs can induce hypersensitivity, which may further aggravate the susceptibility and severity of allergic-like reactions. Barbosa et al. first reported a prospective randomized clinical trial based on 3206 adult outpatients at a cancer center [67]. The fasting group fasted for at least 4 h, and 1.5% of patients developed ADR. In the non-fasting group, with a light meal (e.g., juice, jelly, biscuits and sandwiches) within 1 h prior to examination, 0.9% of patients developed ADR. There was no significant difference in the frequency of symptoms between the two groups. The most common manifestations were nausea, weakness, and vomiting. In addition, although not common, some unexpected symptoms (e.g., flushing, ear itching, dizziness, tremor, tingling, tachycardia, and headache, pain at the injection site) occurred more frequently in the fasting group, which were likely to cause irritability and noncooperation. Encouragingly, the abdomen scan images of 1210 cases were evaluated, and all of them were of adequate diagnostic quality to not require repeated scans. These results suggested that the preparation procedures prior to examinations could be simplified, declining the inconvenience and discomfort of cancer patients.

Very recently, Neeman et al. reported a randomized controlled trial (ClinicalTrials.gov: NCT03533348) [69], enrolling 2091 hospitalized patients who underwent non-emergency CECT examinations. Among them, 1080 cases were allocated to the fasting group and fasted for at least 4 h, and 1011 cases were allocated to the non-fasting group with unrestricted consumption of liquids and solids. The results showed that no case of aspiration pneumonia was identified within 96 h after examination. There was no statistical difference in the frequency of nausea (6.6% vs 7.6%) and vomiting (2.6% vs 3.0%) between the fasting and non-fasting groups. The subgroup analysis results of 1257 patients receiving oral ICM showed no statistical difference in the frequency of nausea (6.8% vs 8.0%) and vomiting (2.6% vs 3.6%) between the fasting and non-fasting groups. The authors concluded that withholding fasting orders was not associated with increased risk of aspiration pneumonia or increased incidence of emetic complications. The incidence of emetic complications in this study was higher than previous literature [11, 67], but it was consistent with the post-marketing surveillance results of iopromide and one recent report involving LOCM [20, 30]. The prospective observation results of 1112 outpatients showed lower incidence of emetic complications than that in inpatients. It might be attributed to the fact that inpatients were often more susceptible to gastrointestinal adverse events due to analgesia, chemotherapy, advanced cancer and other major diseases [71–73].

## Current challenges and prospects

This feature Review gives a comprehensive and detailed description of recent advancements of dietary preparation prior to CECT. Based on rigorous and extensive literature review, no single instance of vomiting-associated aspiration pneumonia has been identified due to the undemanding compliance with common preparatory fasting recommendations prior to CECT yet. The probability of clinically important aspiration is negligible. The longstanding traditional concern that unrestricted consumption of liquids and solids may increase the risk of aspiration pneumonia is out of proportion to the actual risk and lacks methodologically acceptable evidence. Allowing free ingestion of liquids and solids prior to CECT will not increase the risk of aspiration pneumonia and the incidence of emetic complications. The available corresponding evidence strongly suggest us to reevaluate and update current preparatory fasting policy for more appropriate management of dietary preparedness. Abolishing unnecessary fasting instructions can simplify the preparation procedures and workflow prior to CECT, improve the radiologic care quality and efficiency, avoid unnecessary delays or cancellations of examinations, and save on health care costs. It can also ensure that patients are in a normal metabolic state, improve patient convenience, tolerance, cooperation, satisfaction, comfort, and well-being, and reduce the excessive fasting related risks (e.g., dehydration and hypoglycemia) in special patient populations.

Nevertheless, this does not mean that patients should undergo all CECT examinations without preparatory fasting. Like all clinical decisions, theoretical risks should be weighed against the pros and cons on an individual basis. Possible scenarios in which patients should be recommended to fast include but are not limited to: (i) Specific types of examinations (e.g., CT virtual gastroscopy, CECT of gastric wall lesions, CT enterography, CT virtual colonoscopy, CT colonography, preoperative three-dimensional anatomical reconstruction and intracavitary virtual exploration), examinations of lesions near intestinal wall (*e.g.*, pancreas, acute abdomen, mesenteric angiography). Given that gastrointestinal emptying is very important to the image quality of the gastrointestinal examinations, to reduce the possibility of interference and confusion between gastrointestinal contents and surrounding lesions, preparatory fasting should be cautiously maintained. In general, we must keep in mind that preparatory fasting is vital to gastrointestinal tract examinations, while in other kind of examinations, preparatory fasting is not always required. In addition, food ingestion can cause the gallbladder to shrink due to the bile release, thus affecting the observation of lesions on the gallbladder wall. (ii) Patients with esophageal diseases or gastric emptying disorders (e.g., gastrointestinal obstruction, achalasia of cardia, hiatal hernia, gastroesophageal reflux, gastroparesis, pyloric stenosis, tracheoesophageal fistula), patients at high risk of aspiration (e.g., impaired consciousness, increased intracranial pressure, impaired swallowing reflex, hyperemesis, severe obesity) [7, 74], patients requiring continuous fasting for clinical needs, and patients under general anesthesia or sedation [7, 70]. (iii) Patients who receive oral contrast media for abdominal examinations usually need to drink about 1 L volume of liquids, so they have poor compliance when they are full and are reluctant to eat or drink beforehand. On the other hand, the fullness of the gastric cavity can affect the delivery time of oral contrast media, therefore, such patients should be fasted.

From the extreme "start fasting after dinner the day before" to the latest preliminary exploration, we have already taken a solid first step and come a long way. Given the extremely low incidence of emetic complications, large sample sizes are essential to ensure the validity and robustness of the conclusions. The majority of current studies on dietary preparation prior to CECT are with small sample sizes and heterogeneous populations and lack randomization and adjustment of potential confounding factors. Although some observational series and indirect evidence suggested that some factors were associated with emetic complications, no cause-and-effect relationship has been established. Current preparatory fasting recommendations are largely consensus driven, and there is still much more refinement to be done. It is urgent to establish a universal and easy-to-popularize dietary preparation standard prior to CECT. In the future, more prospective clinical observations, randomized control trials, large-scale multi-center studies, clinical feasibility data, expert and practitioner opinions, and open forum commentaries are needed to achieve international multidisciplinary consensus. Combined with the category of scientific evidence (e.g., supportive, suggestive, equivocal, insufficient) or opinion evidence (e.g., expert, membership, informal), to elucidate: (i) the causal relationship between the exact time interval, amount, kinds and content of dietary ingestion and the incidence and severity of emetic complications; (ii) the risk factors that affect the incidence and severity of emetic complications induced by different types of ICM; (iii) individualized optimal dietary preparation protocols for special patient populations (e.g., diabetes, tumor radio-chemotherapy, ultra-young patients). For patients with relatively contraindications to dietary ingestion, early risk stratified assessment should be performed based on evidence-based factors (e.g., patient characteristics, comorbidities, and other clinical variables), to evaluate the risk–benefit for specific patients and formulate structured graded dietary management recommendations. (iv) the preventive effect of pre-medication (*e.g.*, gastrointestinal stimulants, gastric acid secretion inhibitors, antacids, antiemetic drugs, anticholinergic drugs, histamine antagonists, sedative drugs) on the incidence and severity of emetic complications. Furthermore, standardized whole process management should be implemented prior to, during and after examination, to further reduce the incidence of emetic complications and minimize the discomfort of patients.

As a concluding remark, we believe that changes in current preparatory fasting policy prior to CECT will make positive and significant implications on clinical practice. We should strive for evidence-based practice, which requires interdisciplinary cooperation of practitioners covering a diverse range of specialties and practice settings. Practitioners and policymakers should raise awareness, continuously improve the medical care quality, strengthen evidence-based communication with the radiology department, and stress the significance of following these instructions. Through health advertisement and education and combination with specific clinical circumstances, explicit, flexible, and personalized dietary preparation plans could be designed. This would ensure both patient safety and good image quality, which is of great importance to achieving optimal radiological nursing practice and patient clinical benefits, and ensuring efficient, reasonable, safe and normative ICM usage.

## Data Availability

Data sharing is not applicable to this article as no datasets were generated or analyzed during the current study.

## Abbreviations

ACR: American Committee of Radiology
ADR: Adverse drug reactions
CECT: Contrast-enhanced CT
ESUR: European Society of Urogenital Radiology
HOCM: High-osmolality contrast media
ICM: Iodinated contrast media
IOCM: Iso-osmolality contrast media
LOCM: Low-osmolality contrast media
